# Supplementary material for: Quantum 2-Player Games and Realizations with Circuits
Source: Research (Wash D C). 2024 Sep 30;7:0480. doi: 10.34133/research.0480 (PMC11440476; doi:10.34133/research.0480)
Supplement: Supplementary 1 — Sections S1 to S8 Figs. S1 to S8 [file research.0480.f1.docx]

**Quantum Two-Player Games and Realizations with Circuits**

Jinliang Zhang^*^, Tian Chen^*$^, Wenyuan Deng, Xiaoxue Tong and Xiangdong Zhang^+^

Key Laboratory of advanced optoelectronic quantum architecture and measurements of Ministry of Education, Beijing Key Laboratory of Nanophotonics & Ultrafine Optoelectronic Systems, School of Physics, Beijing Institute of Technology, 100081, Beijing, China

*These authors contributed equally to this work. ^+$^Author to whom any correspondence should be

addressed. E-mail: chentian@bit.edu.cn; zhangxd@bit.edu.cn

**Abstract**

Game theory problems are widely applied in many research areas such as computer science and finance, with the key issue being how to quickly make decisions. Here, we present a novel quantum algorithm for game theory problems based on a continuous quantum walk. Our algorithm exhibits quantum advantage compared to classical game algorithms. Furthermore, we exploit the analogy between the wave function of the Schrödinger equation and the voltage in Kirchhoff's law to effectively translate the design of quantum game trees into classical circuit networks. We have theoretically simulated the quantum game trees and experimentally validated the quantum functionality speedup on classical circuit networks. Due to the robust scalability and stability inherent in classical circuit networks, quantum game trees implemented within this framework hold promise for addressing more intricate application scenarios.

1. **Introduction**

Game theory is a collection of mathematical models that study decision-making in situations involving conflict and cooperation, with the aim of abstracting key elements of various competitive scenarios and scientifically investigating their characteristics [1-5]. Recent research has integrated artificial intelligence into game theory problems, combining machine learning methods to excel in domains such as chess and Go. This convergence has resulted in the development of high-performance computer programs capable of playing at a superhuman level [6-13]. A typical example in the game theory is the two-player game, which involves the decisions from two agents. This problem can be cast into a decision tree, to determine which agent wins by calculating the optimal value function within this tree. This is accomplished through high-performance alpha-beta search techniques, which efficiently explore vast search spaces, or by utilizing a general-purpose Monte Carlo Tree Search (MCTS) algorithm. These methods allow researchers to subsequently provide the optimal solution. In such scenarios, the ability to make decisions quickly often proves to be a decisive factor in achieving success. Research has shown [14] that if two-player game trees are viewed as AND-OR trees, then the minimax value of the game tree corresponds precisely to the value of the optimal solution in the AND-OR tree. Therefore, the evaluation of game problems hinges on efficiently obtaining the optimal solution to the AND-OR tree problems. In the assessment of AND-OR tree problems [15-17], classically, the value of a balanced binary AND-OR tree with zero-error in expected time $O(N^{\log_{2}[(1+\sqrt{33})/4]})=O(N^{0.754})$ can be computed using a technique called alpha-beta pruning. Despite the fact that no algorithm has outperformed this classical zero-error algorithm for a long time, it remains quite slow.

On the other hand, quantum algorithms offer significant advantages in addressing specific two-player game problems. The simplest case is the Prisoners’ Dilemma, which is the one-step decision problem. When quantum strategies are employed in the Prisoners’ Dilemma, the decision-making process no longer presents a dilemma [18, 19]. Not only the one-step decision problem, the many-step decision problem is also studied with quantum algorithms. Some works encode all possible solutions of a variety of game search problems into a Hilbert space. By using Grover’s quantum search algorithm [20], they achieve a quadratic speedup over naive classical algorithms [21-24]. Then, the bounded-error quantum algorithms based on quantum circuit model have been developed for evaluating game trees [25-30] on a graph, which requires an $O(\sqrt{N}\log N)$ query for evaluating AND-OR formulas with size $N$. However, it remains unclear how to experimentally implement these algorithms or whether tree structures can be used for this purpose.

In this paper, we propose a novel scheme to realize a two-player game based on AND-OR tree structures. The contributions of our work are two-fold. Firstly, we employe a subgame design technique to develop a quantum algorithm for the Hamiltonian AND-OR tree using continuous-time quantum walk. Our proposed algorithms achieve a query time of $O(\sqrt{N})$ for evaluating preprocessed approximately balanced AND-OR trees. Secondly, we validate the quantum speedup characteristics of this algorithm within circuit networks.

The organization of this paper is as follows. In Section 2, we introduce basic concepts related to game trees and present the theory of quantum two-player games based on tree structures. In Section 3, we demonstrate how to design the quantum algorithm in circuit networks, showcasing its gaming functionality with quantum speedup. The corresponding experimental results of quantum two-player games in circuit networks have been addressed in Section 4. Finally, we discuss and provide future outlooks. The quantum speedup in game tree solving plays a crucial role in various aspects of society, particularly in fields like artificial intelligence and deep learning.

1. **Theoretical Scheme of Quantum** **Two-Player Zero-Sum Games**

In this section, we propose a novel multi-step decision-making framework for a two-player game based on a continuous time quantum walk, demonstrating quantum speedup. Before providing the details of the quantum two-player game, we initiate the process by constructing a quantum AND-OR tree using the quantum walk at first.

- 1. **Quantum AND-OR Tree Based on Quantum Walk**

Here, we demonstrate quantum speedup for the quantum AND-OR tree. First, we design and implement the most common basic two-input OR gate and two-input AND gate structures in the quantum game tree. These two-input OR and AND gates are constructed by using the negative-AND (NAND) gate. The construction details of the NAND gate are shown in S1 of Supporting Information.

The design scheme for the quantum OR tree is shown in Fig. 1a. For a two-input OR gate, the depth of the quantum OR tree is $d=2$. Here, the parameter $L$ is taken as $L=8\sqrt{N}\approx12$ with $N=2$. There are a total of $M=32$ nodes. It includes 25 nodes of the runway, three nodes of the tree structure, and four nodes of the input layer. The top row input has two states, with connections and disconnections between the first two rows of nodes corresponding to inputs 1 and 0, respectively. The lowest root node in the tree structure is connected to a runway at the node $L\text{+1=13}$, in which the length of runway is $2L+1=25$. Each node in Fig. 1a can be represented by a $32\times1$ dimensional quantum state, where the state function $|r\rangle=(0,...0,1,0,...,0)^{T}$ represents that the $r$-th element of the column vector is 1, and all other elements are 0. Thus, the entire quantum OR tree can be represented by a $32\times32$ dimensional Hamiltonian $H$. The details of constructing a quantum OR tree are provided in S2 of Supporting Information.


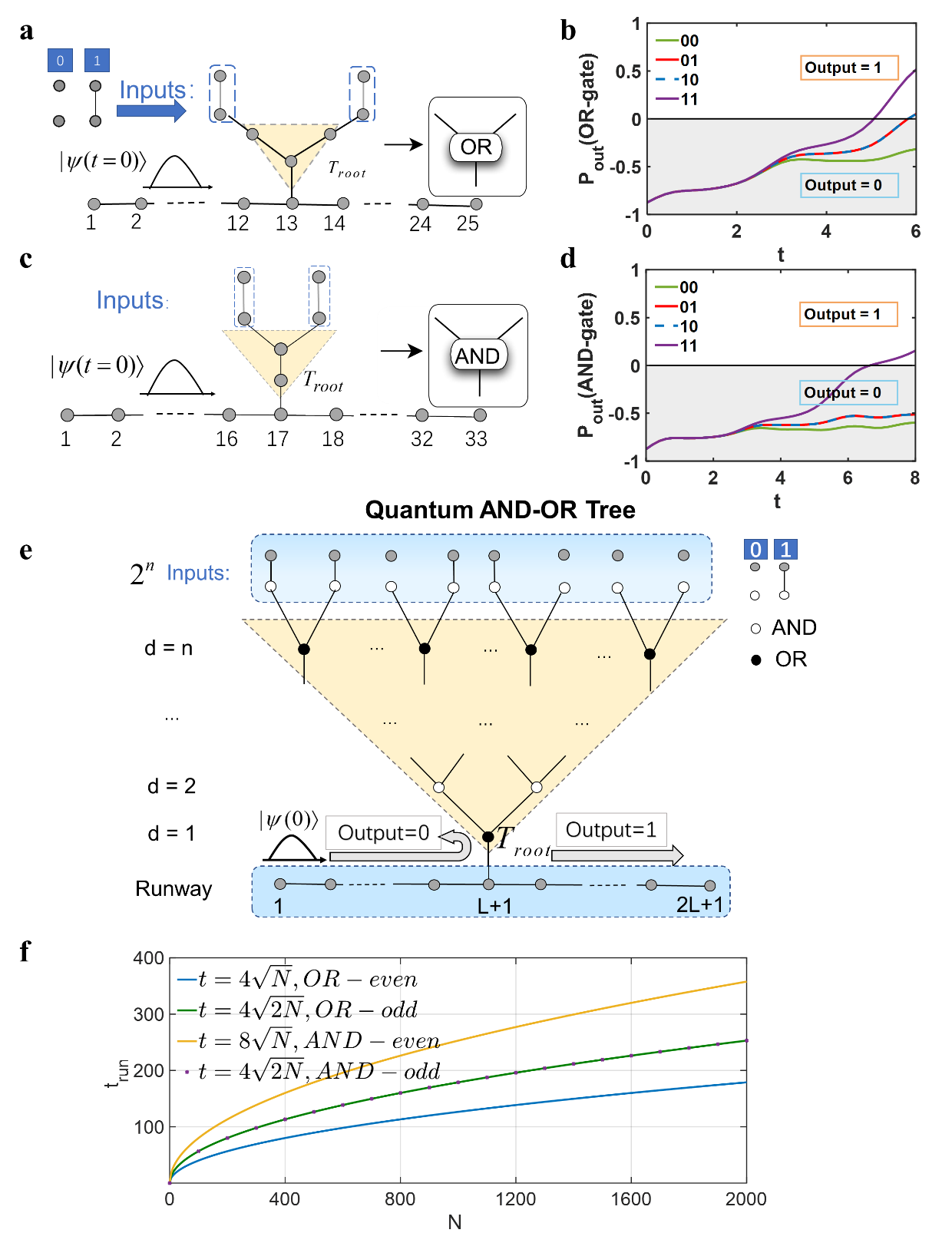


**Figure 1. a)** **Diagram for the two-input quantum OR tree. If the nodes within the blue dashed box at the top are connected (disconnected), the input is 1 (0). b) The output results of the quantum OR tree. The result is 0 when the input is 00, and it is 1 for inputs 01, 10, and 11, respectively. c) Diagram for the two-input quantum AND tree. d) The output result of the quantum AND tree. The output is 0 when the input is 00, 01, and 10, and it is 1 only for the input 11. e) Schematic diagram of the quantum AND-OR tree. With a depth of $n$, the input layer has $N=2^{n}$ inputs, where the connections and disconnections between the nodes of the input layer are represented by 1 and 0, respectively; white nodes represent the AND nodes, while black nodes represent the OR nodes. As the depth of the tree increases, the nodes alternate between black and white. The root node ($T_{root}$) of the tree structure is the OR node, which is connected to a runway of length $2L+1$, and the initial $|\psi(0)\rangle$ is input from the left side of the runway. f) The relationship between the output result time $t_{run}$ and the input parameter $N$ for the four different structures of AND-OR trees. These trees are classified by their root nodes (AND nodes or OR nodes) and their depth (odd or even layers). All of them exhibit quantum speedup.**

In our study, at time $t$, the wave function of the system can be represented as $\psi(t)=(\psi_{1}(t),\psi_{2}(t),\cdots,\psi_{32}(t))^{T}$. We construct the initial state of the system at time $t=0$, $|\psi(0)\rangle=\frac{1}{\sqrt{L}}\sum_{r=2}^{r=13} e^{ir\pi/2}\text{|}r\rangle$ [31, 32], as shown in the bottom left corner of Fig. 1a, the initial state distribution is located on the left side of the bottom runway. At time $t$, the state function evolves into $|\psi(t)\rangle=e^{iHt}|\psi(0)\rangle$, and $t_{run}\text{=}\frac{L}{2}\text{= 6}$. During the evolution process, we focus on the probability of the initial wave packet appearing on either side of the bottom runway. If the probability of the wave packet appearing on the left runway is greater than that on the right runway, the root node outputs 0. Conversely, if the probability of the wave packet appearing on the right runway is greater than that on the left runway, the root node outputs 1. To better illustrate the computation results, we define the output as $P_{out}\text{=}P_{\in R}-P_{\notin R}$​, where $P_{\in R}=\sum_{L+1<r<2L+2} |\langle r|\psi(t)\rangle|^{2}$ and $P_{\notin R}=\sum_{r\notin[L+2,2L+1]} |\langle r|\psi(t)\rangle|^{2}$. It can be observed that when $P_{out}>0$ , the quantum OR tree evaluates the root node as 1, and when $P_{out}<0$, the quantum OR tree evaluates the root node as 0. Detailed derivations can be found in S2 of Supporting Information.

Due to the symmetry of the two-input quantum OR tree structure, it effectively encompasses three distinct inputs: 00, 01 (or 10), and 11. The temporal evolution of the calculation results $P_{out}$ for the two-input quantum OR tree is depicted in Fig. 1b. The various curves in the Fig. 1b represent the evolution of the output of the root node over time for the input scenarios 00, 01, 10, and 11, respectively. It is evident that after time $t>6$, the output results for inputs 01 (red solid line), 10 (blue dashed line), and 11 (purple solid line) are $P_{out}>0$, which indicates that the wave packet is more likely to occupy nodes on the right side of the runway. In this scenario, the output of the root node of the tree is 1. In contrast, for the input scenario of 00 (green solid line), $P_{out}$ is always less than 0. This means that the wave packet is more likely to occupy nodes on the left side of the runway and within the tree, consequently leading to an output of 0 at the root node. That is, we have realized a basic two-input quantum OR tree.

Similarly, we can also construct a two-input quantum AND tree, as shown in Fig. 1c. The depth of the quantum AND tree is $d=2$, where $L$ is chosen as $L=8\sqrt{2N}=16$, there are a total of $M=39$ nodes. It includes 33 nodes of the runway, two nodes of the tree structure, and four nodes of the input layer. The first two rows of nodes also have two states, representing the values of inputs 1 and 0, respectively. The root node of the tree is connected to a runway at the node 17, in which the length of runway is $2L+1=33$. Thus, the entire quantum AND tree can be represented by a $39\times39$ dimensional Hamiltonian $H$. Details for the construction of quantum AND tree have also been shown in S2 of Supporting Information. In our study, at time $t$, the wave function of the system can be represented as $\psi(t)=(\psi_{1}(t),\psi_{2}(t),\cdots,\psi_{39}(t))^{T}$. In such a case, the initial state of the system at time $t=0$ is expressed as $|\psi(0)\rangle=\frac{1}{\sqrt{L}}\sum_{r=2}^{r=17} e^{ir\pi/2}\text{|}r\rangle$ [31, 32], its distribution is located on the left side of the bottom runway. At time $t_{run}\text{=}\frac{L}{2}\text{=8}$ , we can obtain the output of the AND tree. The curves in Fig. 1d represent the change of $P_{out}$ over time for inputs of 00, 01, 10, and 11, respectively. It can be seen that for the input scenario of 11 (purple solid line), after time $t>8$, $P_{out}>0$, resulting in the output of the tree being 1. In contrast, for the other input scenarios (00, 01, 10), $P_{out}$ is always less than 0, leading to the output of the root node of the tree being 0. This approach allows us to implement a fundamental two-input quantum AND tree.

Based on the two-input quantum AND tree and OR tree, we construct a general quantum AND-OR tree as shown in Fig.1e, comprising three main components: the input layer, the AND-OR tree structure, and the runway. In the input layer (the light blue area in Fig. 1e), connections and disconnections between the two rows of nodes correspond to inputs of 1 and 0. They are then processed within the AND-OR tree structure (the light yellow area in Fig. 1e). Consequently, the result of the root node is considered as the output of the tree structure. The root node in the bottom layer of the tree structure is connected to a runway of length $2L+1$ (the dark blue area in Fig. 1e), where there are $L$ nodes on each side of the connected point. The nodes on the runway are numbered from 1 to $2L+1$, while the nodes of the tree structure are sequentially numbered from the bottom to the top. Here, we set the value of $L$ to be $L=8\times2^{n/2}$ for the quantum AND-OR tree structure of depth $n$, there are a total of $M=3\times2^{n}+2^{n/2+4}$ nodes. Each node in Fig. 1e can be represented by a $M\times1$ dimensional quantum state, where the state function $|r\rangle=(0,...0,1,0,...,0)^{T}$ represents that the r-th element of the column vector is 1, and all other elements are 0. Thus, the entire quantum AND-OR tree can be represented by a $M\times M$dimensional Hamiltonian $H$. The details for the construction of the quantum AND-OR tree have also been presented in S3 of Supporting Information.

Similarly to the quantum OR tree, the wave function of the system at time $t$ can be represented as $\psi(t)=(\psi_{1}(t),\psi_{2}(t),\cdots,\psi_{M}(t))^{T}$. The initial state of the system at time $t=0$ is $|\psi(0)\rangle=\frac{1}{\sqrt{L}}\sum_{r=2}^{r=L+1} e^{ir\pi/2}\text{|}r\rangle$ [31, 32], as shown in the bottom left corner of Fig. 1e, where the initial state distribution is located on the left side of the bottom runway. The initial state evolves according to the Schrodinger equation: $i\frac{d}{dt}\psi(t)=H\psi(t)$. We focus on the output result $P_{out}\text{=}P_{\in R}-P_{\notin R}$ at the time $t_{run}\text{=}\frac{L}{2}$.

After discussing the fundamental functionalities of the two-input AND tree and OR tree alongside the quantum AND-OR tree, we proceed to a detailed analysis of their quantum speedup properties. Assuming that all AND-OR trees are of a two-input nature, four distinct structures emerge based on the characteristics of the root node (AND or OR) and the depth level (odd or even).

When the root node of an AND-OR tree is an OR node and the tree depth is even (where $n$ represents the depth of the tree, $n=2k,k=0,1,2...$), the corresponding quantum walk describing the tree (with depth denoted by $d$) remains unchanged, that is, $d=n=2k$. However, when the tree depth is odd ($n=2k+1,k=0,1,2...$), an additional layer of depth is added to the input layer of the corresponding quantum game tree, resulting in a tree depth of $d=n+1=2k+2$. Similarly, if the root node is an AND node, and tree depth is even ($n=2k,k=0,1,2...$), both the root node and the input layer of the corresponding quantum tree increase by one layer, resulting in a depth of $d=n+2=2k+2$. In the case of odd ($n=2k+1,k=0,1,2...$) depth scenarios, the corresponding quantum tree adds one layer at the root node, yielding a tree depth of $d=n+1=2k+2$. Detailed descriptions of these four types of AND-OR tree structures based on quantum walk have been presented in S4 of Supporting Information.

Fig. 1f illustrates the relationship between the output result time $t_{run}$ and the input parameter $N$ for these four types of structures. It is noted that this output time corresponds to the duration required for fully distinguishing different results, and the output result in 0 or 1 does not change with a longer time evolution. We examine the evolution trend of theoretical values with changes in the input scale $N$ for different structures. For each structure, we can conclude that under the condition of input scale $N$, the output time satisfies $t\propto\sqrt{N}$with $N$, thereby demonstrating the quantum speedup.

- 1. **Description of** **Two-Player Zero-Sum Games**

As shown in the previous section, the quantum speedup in the quantum AND-OR tree has been demonstrated. In the following, we give the description of two-player zero-sum games at first; then we map this game problem to the quantum AND-OR tree, and show the quantum speedup.


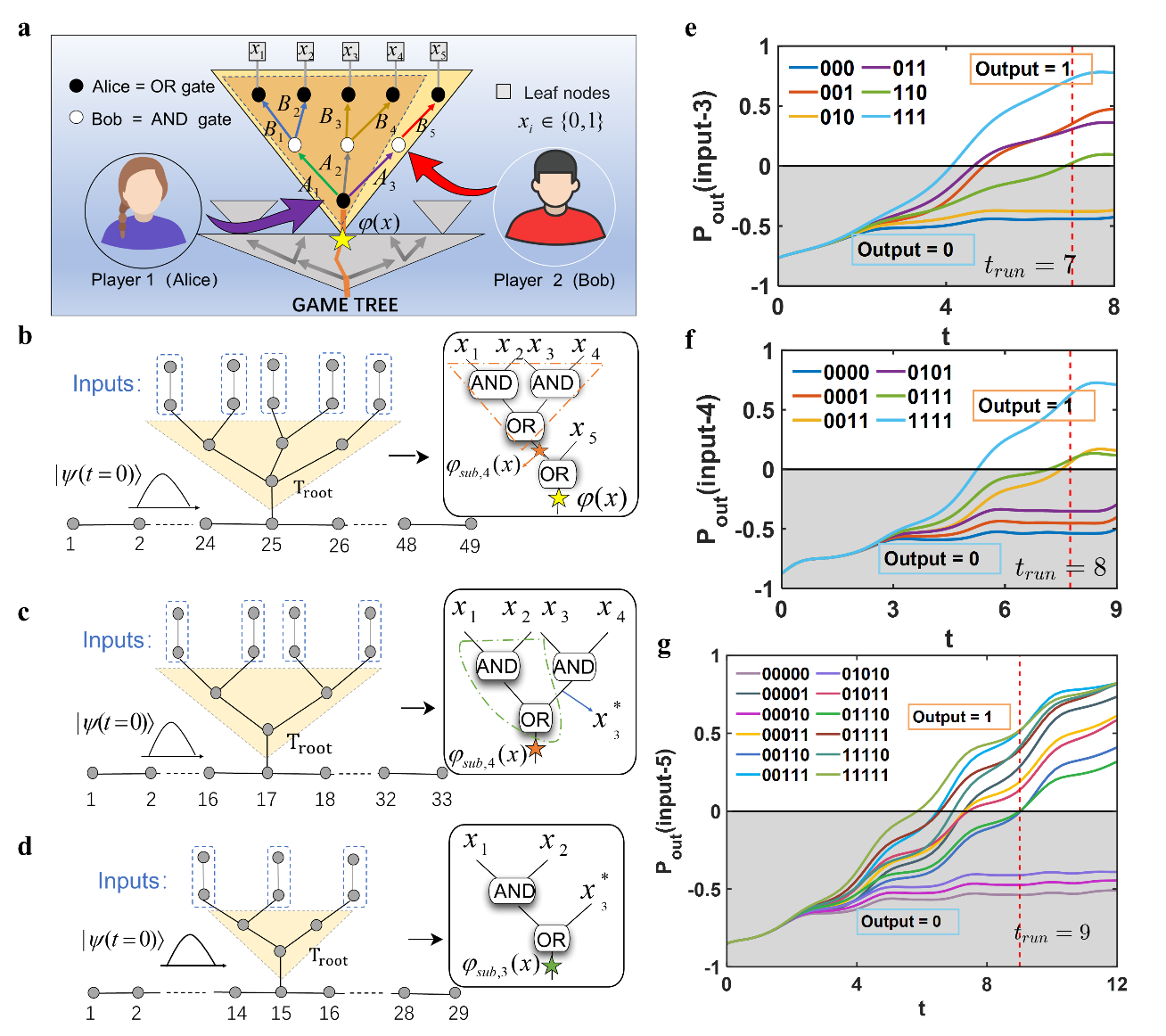


**Figure 2. a) Alice and Bob double zero-sum game tree. The entire game tree is made up of numerous subtrees. Each gray triangle represents a subtree, while the orange-yellow line depicts the optimal path throughout the entire gameplay process. The black nodes represent the choice of Alice (OR node) and the white nodes represent the choice of Bob (AND node). The input values of the terminal nodes $x_{i}$ are only 0 and 1. The green arrows denote the strategies available to Alice, with the strategy $A_{1}$, $A_{2}$, or $A_{3}$ for a particular node in the game process. In contrast, the blue arrows represent the strategies available to Bob, who can choose strategy $\{A_{1}|B_{1},B_{2}\}$, $\{A_{2}|B_{3},B_{4}\}$, and . The dashed brown triangles and the solid yellow triangles represent the four-input subtree and the five-input subtree, respectively. b) Schematic diagram of the five-input quantum AND-OR tree. On the right side is the corresponding classical game tree structure. c) Schematic diagram of the four-input quantum AND-OR tree. d) Schematic diagram of the three-input quantum AND-OR tree. e)** **Results for a three-input quantum AND-OR tree. The inputs 000 and 010 result in an output of 0, while the inputs 001, 011, 110, and 111 lead to an output of 1. f) Results for the four-input quantum AND-OR tree. The input 0000, 0001, and 0101 result in the output of 0. The inputs 0011, 0111, and 1111 result in an output of 1. g) Results for the five-input quantum AND-OR tree. The inputs 00000, 00010, and 01010 result in an output of 0. All other inputs result in an output of 1. The red dashed line represents the measurement time $t_{run}$.**

The game process is typically represented by the game tree illustrated in Fig. 2a. During the game, the first player (Alice) makes the “OR” operation of their child nodes, and the second player (Bob) does the “AND” operation of their child nodes. These are labeled by the black and white dots respectively in Fig. 2a. One round of game contains one “OR” and “AND” operations. In one round, Alice can make a choice at the beginning, and Bob wants to choose the strategy to win, no matter what moves made by Alice before. In this way, a new round of game is run. Alice needs to find a new strategy to win in the new situation, and the “OR” operation is made by Alice again. Therefore, the “OR” and “AND” operations (black and white dots in Fig. 2a) appear alternatively during the game process. The arrows in different colors in Fig. 2a represent different choices. When Alice chooses the decision $A_{1}$ (the green arrow in Fig. 2a), Bob can choose between $B_{1}$ and $B_{2}$ (blue arrows); when Alice chooses $A_{2}$ (grey arrow), Bob can choose between $B_{3}$ and $B_{4}$ (gold arrows); and when Alice chooses $A_{3}$ (purple arrows), Bob can only choose $B_{5}$ (red arrows). The game is terminated when reaching terminal leaf nodes (rectangles in the top of Fig. 2a). Each leaf node contains a variable $x_{i}$with the value of 1 or 0. Based on the inputs of the terminal leaf nodes, we can quickly determine the decision paths of Alice and Bob, and evaluate the output result of the root node (the yellow star in Fig. 2a) of the game tree. In the game between Alice and Bob, Alice wins if the root node value equals 1; conversely, Bob prevails when the root node value is 0.

The game problem addressed in Fig. 2a can be mapped to a five-input quantum AND-OR tree, as illustrated in Fig. 2b, which is composed of dashed brown subtrees representing a four-input subtree (the light-yellow solid subtree in Fig. 2a) and a two-input OR tree. Now, we employe a subgame design technique to develop a quantum algorithm for the Hamiltonian AND-OR tree using continuous quantum walk, and then realize the quantum speedup in the gameplay process with a five-input quantum AND-OR tree. The formula representation of this tree is as follows: , where  and  denote AND nodes and OR nodes, respectively. The five input values are $x_{1}$, $x_{2}$, $x_{3}$, $x_{4}$, and $x_{5}$; represents the result of the calculation of the root node of the five-input quantum AND-OR tree. Additionally, the AND-OR tree can further decompose into a smaller four-input subtree and a two-input OR tree, . The operational formula for the four-input subtree is as follows:, as shown in Fig. 2b. The four-input subtree can then be further decomposed into a smaller three-input subtree and a two-input AND tree; see the inset of Fig. 2c. The three-input subtree, as depicted in Fig. 2d, is characterized by the following operational formula: , where $x_{3}^{*}$ represents the result of the calculation for the input $x_{3}$and $x_{4}$.

For the three-input subtree, here we take the value of $L$ to be $L=8\sqrt{3}\approx14$. The top row of inputs in Fig. 2d can be in connected or disconnected states, corresponding to input values of 1 and 0, respectively. The lowest root node in the tree structure is connected to a runway of length 29, in which the connection node is 15. This tree structure features six independent inputs: 000, 001, 010 (100), 011 (101), 110, and 111. The results of the calculation of the output of the three-input subtree over time are depicted in Fig. 2e. From the graph, it is evident that after time $t>t_{run}(\frac{L}{2}=7)$, the input types: 110 (green line), 011 (purple line), 001 (orange-red line), and 111 (light blue line) have output $P_{out}>0$. This signifies that under these input types, the wave packets of the input predominantly occupy the right side of the runway, leading to a computation result of 1 at the root node. On the contrary, the results $P_{out}$ for input 000 (blue line) and 010 (orange-yellow line) consistently remain less than 0. This suggests that the wave packets of the input are predominantly reflected onto the left side. Consequently, the corresponding inputs yield a result of 0. This implies that in the game played within this three-input subtree, if the inputs are 110, 011, 001, or 111, Alice wins; whereas if the inputs are 000 or 010, Bob wins.

Furthermore, by integrating the three-input subtree with a two-input AND tree, a larger four-input subtree is obtained, as depicted in Fig. 2c. We take the value of $L$ to be $L=8\sqrt{4}=16$. Due to structural symmetry, there are only six distinct input combinations for the four inputs: 0000, 0001 (0010, 0100, 1000), 0011 (1100), 0101 (1010, 0110, 1001), 0111 (1011, 1101, 1110), and 1111. The time-varying calculation results of the output of the four-input quantum AND-OR tree are shown in Fig. 2f. It is evident that after time $t>8$, the output $P_{out}>0$ for 0111 (green line), 0011 (orange-yellow line), and 1111 (light blue line) indicate that under these input types, the wave packets predominantly occupy the right side of the runway. In these respective input scenarios, the calculation results of the four-input quantum AND-OR tree is 1. For comparison, it is evident from the figure that the output $P_{out}$ for 0000 (deep blue line), 0001 (orange-red line), and 0101 (purple line) consistently remain below 0. Under these input types, the wave packets are predominantly reflected to the left side of the runway, resulting in a corresponding calculation result of 0. This implies that in the larger four-input subtree game, if the inputs are 0011, 0111, or 1111, Alice wins; however, if the inputs are 0000, 0001, or 0101, Bob wins. It should be noted that, compared to the three-input subtree earlier, if Alice wins in the three-input subtree game, then in the four-input subtree game, regardless of the value of the fourth input, Alice will ultimately win.

Compared to the four-input subtree, the final five-input quantum AND-OR tree introduces an additional input while maintaining structural symmetry. There are a total of 12 independent combinations of inputs, namely: 00000, 00001, 00010, 00011, 00110, 00111, 01010, 01011, 01110, 01111, 11110, and 11111. For the five-input AND-OR tree, we take the value of $L$ to be 24. The small deviation away from $8\sqrt{5}\approx18$ is due to the asymmetric input in the middle (Fig. 2b).

The time-varying calculation results of the output for the five-input quantum AND-OR tree are depicted in Fig. 2g. It is noteworthy that the output results $P_{out}$ for 00000 (brown line), 00010 (pink line), and 01010 (purple line) consistently remain below 0. This means that under these input types, the wave packets predominantly concentrate on the left side of the runway, resulting in an output value of 0. In contrast, it is evident from the figure that, after time $t>9$, the output results $P_{out}$ for the remaining nine cases are all greater than 0. Under these inputs, the wave packets predominantly occupy the right side of the runway, resulting in an output value of 1. Similarly to the previous description, for the five-input game tree, if Alice wins in the four-input subgame, regardless of the input of the fifth bit in the five-input game tree, Alice will ultimately win the five-input game.

Therefore, for the three-input subtree, the four-input subtree, and the five-input game tree, from the study above, we can conclude that with the input scale $N$, the time to obtain the output result satisfies $t\propto\sqrt{N}$with $N$, which demonstrates the quantum speedup. Since then we have introduced the design of a two-player zero-sum game tree based on quantum walk and presented the corresponding results. In the following discussion, we explore how to design classical circuit networks to simulate these quantum game trees.

1. **Circuit Designs of Quantum Two-Player Games**

In this section, we provide the circuit design for a general quantum AND-OR tree. As depicted in Fig. 3a, each circle corresponds to a node in the structural diagram shown in Fig. 1e, with a total of $M=3\times2^{n}+2^{n/2+4}$ nodes in the entire circuit structure. Similarly to the numbering of nodes in Fig. 1e, we start by numbering the nodes on the runway and then proceed to number the nodes from the bottom to the top of the tree structure. The voltage states on these nodes can be represented by column vector , where the voltage $V_{i}(t)$ represents the voltage value at the $i$-th circuit node at time $t$. In Fig. 3a, the top two rows of nodes in the circuit are connected via dual in-line package (DIP) switches. These DIP switches can be adjusted to be connected or disconnected, corresponding to input values of 1 or 0, as shown in Fig. 3b. In Fig. 3a, each node is grounded using a capacitor resistor, with the grounding method varying according to the design requirements. The nodes in the circuit network are grounded via capacitors and resistors or negative impedance converter NIC modules [33, 34], as shown in Fig. 3c, d, e, and g. Each node is connected via a negative impedance converter (NIC), as depicted in Fig. 3f. This structure consists of an operational amplifier, two resistors connected to the positive and negative terminals of the operational amplifier, and a resistor $R_{i,j}$ (representing the effective resistance from node $i$ to node$j$). The NIC module can reverse the direction of current flow from node $i$ to node $j$ and from node $j$ to node $i$ [35-37].

Therefore, the Kirchhoff equation set for the $i$-th current node can be expressed as:

$$\begin{matrix} C_{i}\frac{dV_{i}(t)}{dt}+\frac{V_{i}(t)}{R_{i}}=\sum_{j} \frac{V_{j}(t)-V_{i}(t)}{R_{i,j}} \\ \frac{dV_{i}(t)}{dt}=\frac{1}{RC_{i}}\sum_{j} [V_{j}(t)-V_{i}(t)] \end{matrix}$$

Here, the capacitance value $C_{i}$ and the resistance value $R_{i}$ represent the capacitance and grounding resistance values of the $i$-th circuit node, respectively. The node equation set for the entire circuit system can be expressed as:

The matrix $A$ is a real matrix of size $M\times M$, and its elements are determined by the capacitance and resistance between the nodes of the circuit. If we multiply equation (2) by the imaginary unit $i$, we obtain . In our study, we choose the appropriate grounding resistor values so that the diagonal elements of the matrix $A$ are all zero. Then adjust the effective resistance values of the NICs to ensure that the matrix satisfies the condition of being a Hermitian matrix. The Matrix and the Hamiltonian $H$ can be obtained through similarity: , we can get:

In the equation, $P$ represents the similarity transformation matrix, whose specific form is provided in S5 of Supporting Information. If we regard as the wave function $\psi(t)$ in the Schrödinger equation, then the evolution of the circuit system exactly corresponds to the time evolution of the quantum system. It is found that the initial state in circuit evolution is similar to the initial wave function designed in quantum theory, where the voltage unit is set as$\sqrt{L}V^{-1}$.


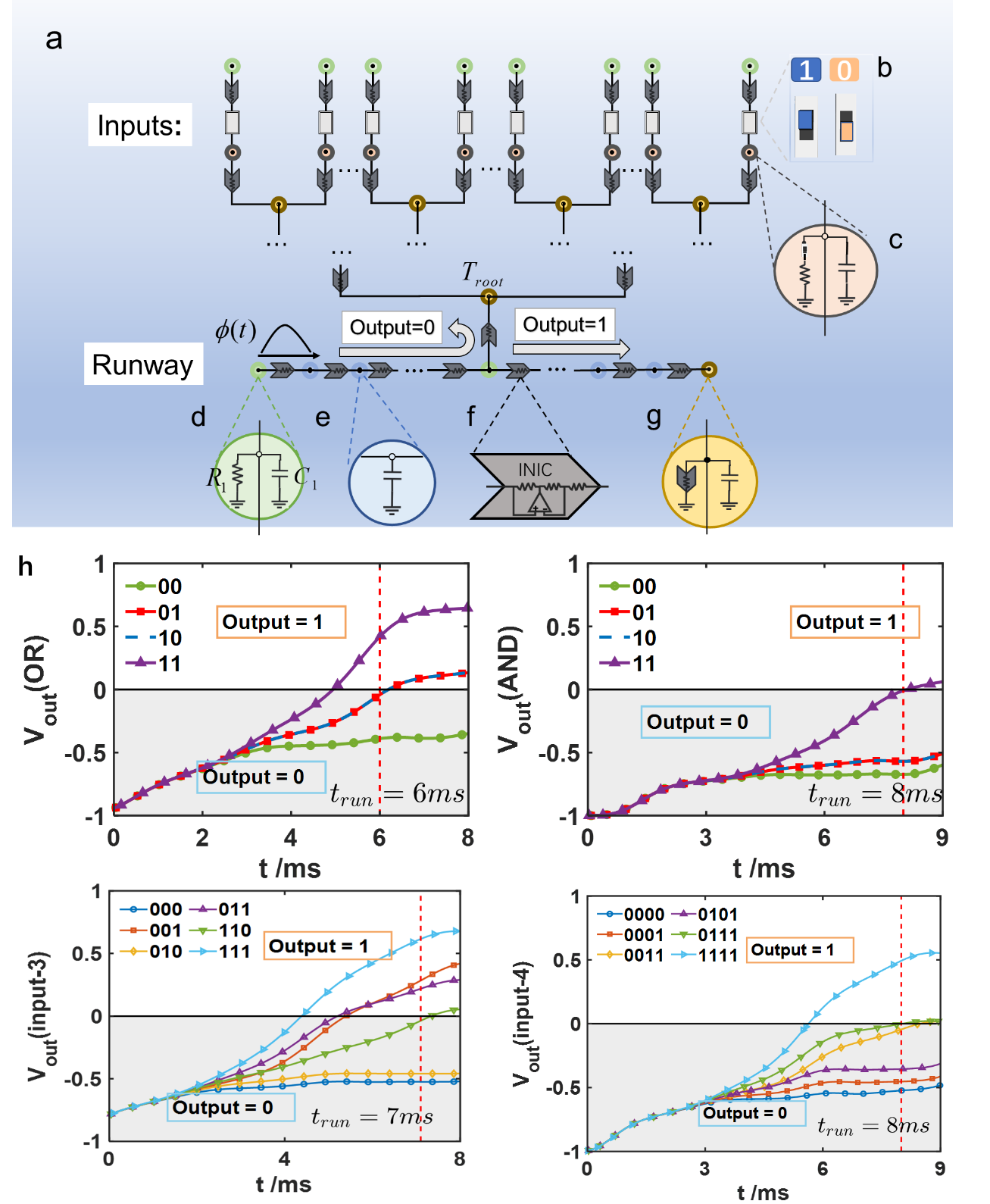


**Figure 3. a) Circuit design of the quantum AND-OR tree. b) The connections and disconnections of the switches at the top represent inputs 1 and 0, respectively. c) The pink nodes in the middle are connected to the ground through capacitors and resistors in parallel. d) When the switch in the pink node is connected to the child node (input is 1), the switch in the pink node should be closed, that is, the capacitor and resistor are in parallel with the ground. e) When the switch is open, the pink node should only be disconnected from the capacitor and connected to the ground. f) Schematic diagram of the negative impedance converter (NIC), which includes an operational amplifier, two positive and negative resistors, and an equivalent resistor. g) Nodes are connected to the ground in parallel through NIC modules and capacitors. h) Simulation results of the two-input quantum OR tree, the two-input quantum AND tree, the three-input AND-OR tree, and the four-input AND-OR tree, which are consistent with the theoretical calculations in Fig. 2.**

At the initial moment, 1 V DC voltage is applied to the corresponding $L$ nodes on the runway left side, with the remaining nodes grounded. After the evolution begins, we disconnect the initial voltage of 1V and grounding from the remaining nodes. The voltage state evolves according to Kirchhoff's current law. During the evolution of the circuit, the operating time $t_{circuit}$ of the circuit equations differs from the theoretical operating time $t_{theory}$ of the system by only one constant term: $t_{circuit}=t_{theory}*(R_{i}C_{i})$ [32, 38]. In our simulations and experiments, the resistance $R_{i}$ is set to 10kΩ, and the capacitance $C_{i}$ is set to 100nF. Therefore, in experiments, 1ms corresponds to a theoretical time unit of 1. The output result can also be represented by subtracting the voltage of the remaining nodes from the voltage at the right end of the runway, that is $V_{out}(t)=\sum_{i\in R} \left|V_{i}(t)\right|^{2}-\sum_{i\notin R} \left|V_{i}(t)\right|^{2}$. This way, by measuring the voltage values of circuit nodes at different times using an oscilloscope, we can determine the computation result of the quantum AND-OR tree.

In Fig. 3h, we present the circuit simulation results of the two-input quantum OR tree, two-input quantum AND tree, three-input subtree, and four-input subtree relevant to the game process. The design diagrams of the corresponding circuit structures are included in S6 of Supporting Information. For the two-input quantum OR tree, the results indicate that at time $t>6ms$, the output is 1 ($V_{out}>0$) only when the inputs are 11 (purple upward-pointing triangle line), 10 (blue dotted line), and 01 (red square line), while for all other inputs, it remains 0 ($V_{out}<0$). This simulation result aligns with the theoretical results in Fig. 1b. For the two-input quantum AND tree, at time $t>8ms$, the output is 1 ($V_{out}>0$) only when the input is 11 (purple triangle upwards-pointing line). This simulation result is also consistent with the theoretical results in Fig. 1d. In the three-input subtree, the formula is represented as:, it is only after time $t>7ms$ that the output is 1 when the inputs are 001 (orange square line), 011 (purple upward-pointing triangle line), 110 (green downward-pointing triangle line), and 111 (blue rightward-pointing triangle line). For the four-input subtree, the formula is: , at time $t>8ms$, the output is 1 ($V_{out}>0$) only when the inputs are 0011 (yellow diamond line), 0111 (green pointing downward triangle line), and 1111 (blue pointing rightward triangle line). The circuit simulation results for the three-input subtree and four-input subtree correspond to the quantum theoretical results (see Fig. 2e and 2f). Similarly, for the more complex quantum AND-OR tree structure, we can also utilize the classical circuit designs mentioned above. In S6 of Supporting Information, we also provide the circuit design and results for the five-input game tree. Upon completing the circuit design and simulation, we then proceed to discuss the experimental realization of the quantum game tree circuit.

1. **Experimental Realizations of Quantum Two-Player Games**

Based on the aforementioned circuit designs, we discuss how to proceed with experimental implementation. First, we demonstrate the experimental implementation of a two-input quantum OR tree. In Fig. 4a, the physical printed circuit board (PCB) of the two-input quantum OR tree is displayed. The size of the board is $35cm\times25cm$, the circuit structures prepared for the experiment correspond one to one with the theoretical design depicted in Fig. 3a.

In Fig. 4a, $a_{1}$ denotes push button switches used to control the operation of relays, while $a_{2}$ represents DIP switches. The switches in the open position signify input 0, while they in the closed position signify input 1. The $a_{3}$ represents relays (model G6K-2F-Y-5VDC), used to simultaneously disconnect each node after applying the initial voltage, allowing the system to evolve from the initial time. The $a_{4}$ represents two negative impedance converters (NICs), used to connect adjacent nodes. The capacitors are 100nF and the resistors are 10kΩ. The capacitance and resistance parameters chosen for the experiment determine the values of the circuit matrix. The $a_{5}$ is a pin header used to input the initial voltage and the voltage values of the complex impedances to the circuit board. The silver dashed box represents the runway, the blue dashed triangles represent the quantum OR tree, and the red dashed circle represents the root node of the tree.

For the two-input quantum OR tree, during the circuit evolution, the four different inputs (00, 01, 10, 11) can be controlled by opening or closing the DIP switches. Each node is connected to the initial voltage via relays. The initial state of the circuit system is denoted as , where all nonzero voltages are initially on the left side of the runway ($r\leq L\text{+1}$). When the push-button switch is disconnected, the relays are opened, and each node is disconnected from the initial voltage, initiating the system's evolution. During the evolution, voltages gradually appear at the nodes on the right side of the tree in the experiment. Using an oscilloscope to measure the voltage values of the nodes and calculating the difference between the sum of the squares of voltages at the rightmost nodes and the sum of the squares of voltages at non-rightmost nodes ($V_{out}(t)=\sum_{i\in R} \left|V_{i}(t)\right|^{2}-\sum_{i\notin R} \left|V(t)_{i}\right|^{2}$), we can obtain the output value of the tree's root node.


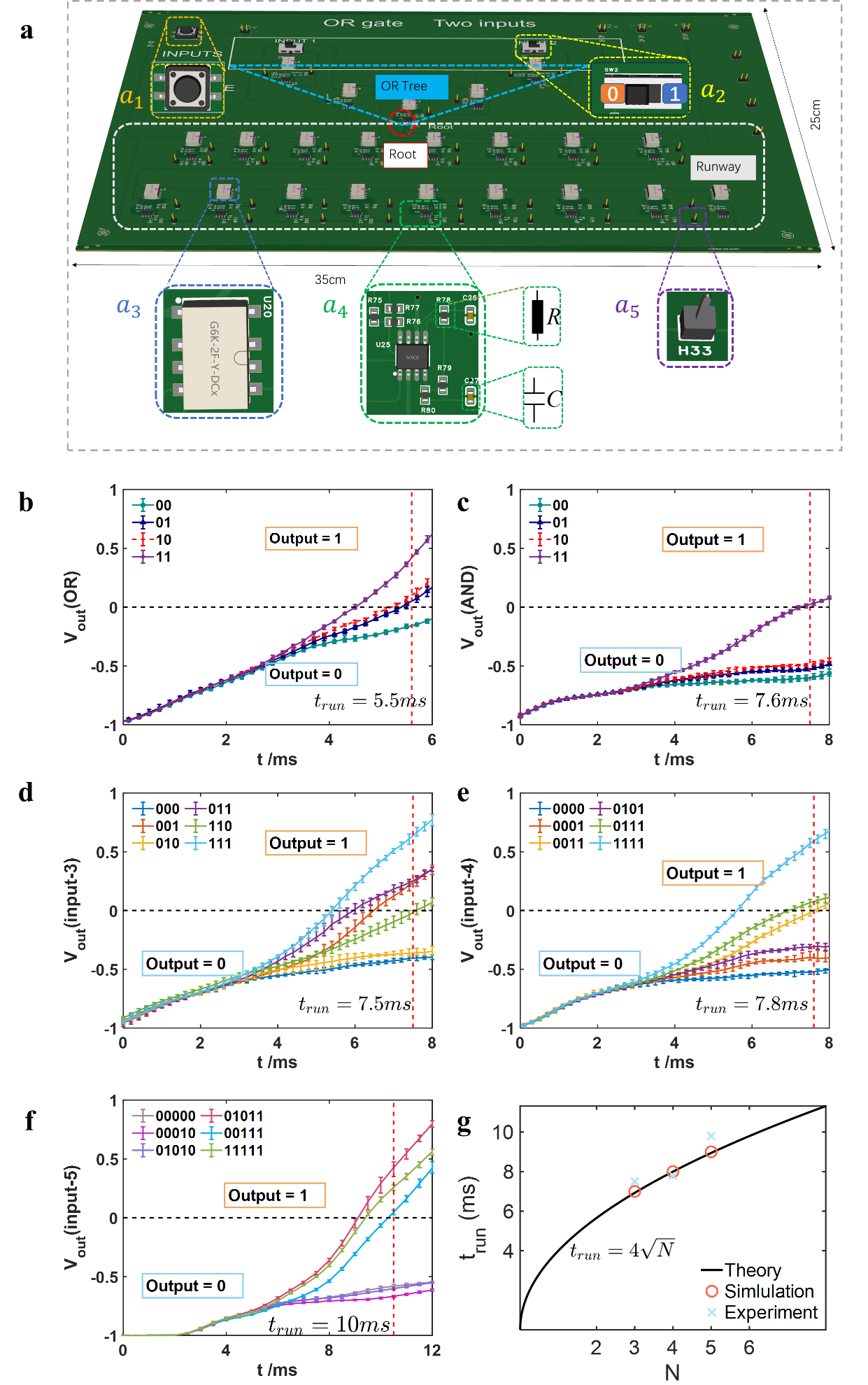


**Figure 4. a)** **Printed circuit board (PCB) of the two-input quantum OR tree structure. Detailed components of the quantum OR tree: $a_{1}$) Button switch to control relay operation; $a_{2}$) DIP switch to select inputs (left for 0, right for 1), providing three independent inputs: 00, 01 (or 10), and 11; $a_{3}$) Relay (model: G6K-2F-Y-5VDC) to control the initial state of the circuit board; $a_{4}$) Two negative impedance modules (LT1013) within the gray box; resistor and capacitor highlighted with green dashed lines; $a_{5}$) Pin headers to introduce initial voltages and provide impedance to the system, as well as for voltage measurements at each node. b) Experimental results of the two-input quantum OR tree: 00 (green line), 01 (blue line), 10 (red dashed line), and 11 (purple line) are depicted. Each line is accompanied by error bars representing the results obtained from five experiments. c) Experimental results of the quantum AND tree of two-input. Inputs are denoted by line colors similar to those in Fig. 4b. d) Experimental results of the three-input quantum AND-OR tree: Among the outputs, 000 (blue line) and 010 (orange-yellow line) are 0. For other inputs, 001 (orange line), 011 (purple line), 110 (green line), or 111 (light blue line), the output is 1. e) Experimental results of the four-input quantum AND-OR tree. The outputs for 0000 (blue line), 0001 (orange-yellow line), and 0101 (purple line) are 0; all other output results are 1. f) Experimental results of the five-input quantum AND-OR tree. The outputs for 00000 (gray line), 00010 (pink line), and 01010 (purple line) are 0; for other inputs such as 01011 (red line), 00111 (blue line), and 11111 (green line), the output results are all 1. g) The relationship between the input $N$ and the output time $t$ for the three-input subtree, the four-input subtree, and the five-input game tree. The solid line represents the theoretical calculation value, the red circle represents the simulated time, and the blue cross represents the experimentally measured time.**

In Fig. 4b, the experimental results for the two-input quantum OR tree are depicted, where green, blue, red, and purple represent the evolution of output node voltages in the circuit structure for inputs 00, 01, 10, and 11, respectively. Each line accompanied by error bars represents the results averaging five times in experiments (the following results in Figs. 4c-4f are similar). It can be observed that after time $t>5.5ms$, an output result of 1 ($V_{out}>0$) is obtained only when the input is 01 (10) or 11, while the other inputs remain at 0 ($V_{out}<0$). It is noted that components in the circuit network, such as capacitors, resistors, and operational amplifiers, may have inherent errors, whereas the LTspice simulation software employs ideal components. Consequently, there may be slight discrepancies in time between simulation and experiment results. And the experimental evolution results are nearly in agreement with the theoretical simulations. We have discussed the experimental error in S7 of Supporting Information. Nevertheless, we have observed that these variances fall within the expected range. This means that the experimental results are reliable.

In Fig. 4c, the experimental results for the two-input quantum AND tree are illustrated, where green, blue, red, and purple represent the evolution of voltages in the circuit structure for inputs 00, 01, 10, and 11, respectively. It can be observed that after time $t>7.6ms$, an output result of 1 is obtained only when the input is 11, while the other inputs remain at 0. Such input results indeed correspond to the functionality of the AND tree. Furthermore, Fig. 4d presents the experimental results for the three-input subtree. The deep blue, orange-red, orange-yellow, purple, light green, and light blue solid lines represent the evolution of voltages in the circuit structure for inputs 000, 001, 010, 011, 110, and 111, respectively. It can be observed that after time $t>7.5ms$, an output result of 1 is obtained only when the inputs are 001, 011, 110, or 111, while for other inputs, the output remains at 0. We also present the experimental results for the four-input subtree and the five-input game tree in Figs. 4e and 4f. For the four-input subtree, after time $t>7.8ms$, an output result of 1 is obtained only when the inputs are 0011 (yellow line), 0111 (green line), or 1111 (light-blue line), while for other inputs, the output remains at 0. Similarly, for the five-input quantum AND-OR game tree, when the inputs are 00000 (gray line), 00010 (pink line), and 01010 (purple line), the output result at the root node remains at 0 ($V_{out}<0$). Conversely, for other inputs such as 01011 (red line), 00111 (blue line), and 11111 (green line), after time $t>10ms$, the output result is 1($V_{out}>0$). The output results for other inputs are provided in S8 of Supporting Information. The experimental results described above correspond well to the circuit simulation results shown in Fig. 3h.

To showcase the quantum speedup of the AND-OR tree in circuit realization, we analyze the time required to distinguish between different outputs based on the tree's input. Specifically, we select the root node as the OR node and set the depth of the AND-OR tree to be even. The relationship between the input $N$ and the output time $t$ is provided in Fig. 4g. According to the quantum theory above (Section 2), it needs the time as $t\propto\sqrt{N}$ with the input bit number, which has been depicted as the solid black line in Fig. 4g. These theoretical results are obtained from the three-input subtree, the four-input subtree, and the five-input game tree in Fig. 2. Here, the unit of time is chosen as millisecond, which corresponds to our circuit design. As shown in our circuit simulation (Fig. 3) and experiment (Fig. 4), the quantity $P_{out}$ has been chosen to show the distribution of probability on the runway. For all cases showing the output of 1, we choose the critical time at which all $P_{out}$ are becoming larger than zero. This time is viewed as $t_{run}$. In Fig. 4g, red circles are the simulation results which are obtained from the time for the three-input subtree, the four-input subtree, and the five-input game tree in the circuit simulation in Section 3. Blue navy crosses denote the times from the experiment (Figs. 4d-4f). As shown in Fig. 4g, it can be observed that the theoretical and simulated times strictly coincide with each other. However, due to the relatively small value of $N$, there is a slight discrepancy between the experimental and simulated time. The experimental results are distributed around the theoretic results (blue solid line), which also demonstrates the quantum advantage in our circuit platform.

1. **Discussion and Conclusions**

Currently, the quantum circuit model can process multiple states simultaneously, potentially offering computational speedup in solving complex game problems. However, quantum computing is still constrained by noise and hardware limitations. Although the theoretical proposal of the balanced NAND-gate on the conjugated organic molecular structure is achieved [39], it is not easily implemented in reality due to the hardness in the accurately control of the internal structure of molecules and electron movement. Recent study shows that the balanced NAND-gate algorithm is tested in a photonic waveguide [40], but fixed inputs and a lack of tunability increased resource consumption and limited its application. In this way, the platforms with the conjugated organic molecules or the waveguides are not able to display the complicated functions required in the game problem easily. Compared with other platforms, the circuit has a high maturity and scalability, and can handle two-player game problems stably and efficiently. In addition, the circuit is deterministic and robust in the calculation results, which is suitable for solving game problems that require high accuracy.

In addition, we have successfully implemented an eight-input AND-OR tree and conducted demonstrations on a PCB platform. Indeed, as the number of components increases, the required area of the PCB expands, and the accumulation of component errors increases, thereby affecting the output results. However, by mapping the implementation from a PCB to an integrated circuit (IC) chip, these issues can be effectively addressed. As shown in [41, 42], the extremely low error rates in the chip enable the realization of low parasitic capacitance and inductance, thereby reducing signal distortion and error accumulation, and improving the precision and stability of the circuit. Implementing the game problem using integrated circuit chips offers the following advantages. For instance, the chip, which contains thousands of complementary metal-oxide-semiconductor (CMOS) transistors, is fabricated using a 65 nm CMOS process technology, with a size of $3000\times3500\mu m^{2}$[42]. Based on this component count, it is estimated that up to 100 inputs for the game problem can be realized. Because of these advantages, the implementation of the game problem on integrated circuits can scale up to larger inputs, while keeping the circuit size within the millimeter range.

In this work, we design a quantum algorithm based on a quantum walk and experimentally verify the quantum speedup on a classical circuit platform. We map the two-player zero-sum game to the AND-OR tree and design the quantum AND-OR tree to solve the game problem. In our study, we choose the five-input game tree as an example and divide it into a four-input subtree and a three-input subtree in sequence. Our results verify that the consistency between the optimal path of a subtree and that of the entire game tree. More importantly, the time for obtaining the output result is satisfies $t\propto\sqrt{N}$ with the $N$ inputs, which has the good correspondence to the time in the quantum algorithm. Circuit simulations and experimental results realize a new type of classical computing based on the quantum intermediary construction. Furthermore, the solution for the two-player zero-sum game can be obtained within the time as that from the quantum algorithm. Although we only discuss the two-player zero-sum game problem here, it is also possible to use the concept of the game tree to demonstrate the quantum speedup for more complex game problems, including chess, economics, cybersecurity, computer science, and finance, among others. The quantum speedup in the game problem here provides a new idea for improving computing power in the era of big data and will be widely used in various fields.

**Conflicts of Interest**

The authors declare that there are no conflicts of interest regarding the publication of this article.

**Authors’ Contributions**

J. L. Z. provided the theoretical analysis cooperated with T. C., J. L. Z. performed the experiments and analyzed the data with the help of T. C., W. Y. D., X. X. T., and X. D. Z. X. D. Z. initiated and designed this research project. J. L. Z and T. C. contributed equally to this work.

**Acknowledgements**

This work was supported by the National Key R & D Program of China under Grant No. 2022YFA1404900 and the National Natural Science Foundation of China (12234004 and 12374323).

**Reference**

1. W. F. Lucas, “An overview of the mathematical theory of games,” Management Science, vol. 18, no. 5-part-2, pp. 3-19, 1972.
2. J. Von Neumann and O. Morgenstern, Theory of Games and Economic Behavior, 2nd rev. ed., 1947.
3. C. Ewerhart, “Backward induction and the game-theoretic analysis of chess,” Games and Economic Behavior, vol. 39, no. 2, pp. 206-214, 2002.
4. T. Alkheliwi, et al., “Applying game theory rules to enhance decision support systems in credit and financial applications,” in 2014 Computer Games: AI, Animation, Mobile, Multimedia, Educational and Serious Games (CGAMES), IEEE, 2014.
5. W. Pijls and A. de Bruin, “Game tree algorithms and solution trees,” Theoretical Computer Science, vol. 252, no. 1-2, pp. 197-215, 2001.
6. F.-H. Hsu, Behind Deep Blue: Building the Computer that Defeated the World Chess Champion. Princeton University Press, 2002.
7. R. Coulom, “Efficient selectivity and backup operators in Monte-Carlo tree search,” in International Conference on Computers and Games, Berlin, Heidelberg: Springer Berlin Heidelberg, 2006.
8. L. Kocsis and C. Szepesvári, “Bandit based Monte-Carlo planning,” in European Conference on Machine Learning, Berlin, Heidelberg: Springer Berlin Heidelberg, 2006.
9. R. Coulom, “Computing 'Elo ratings' of move patterns in the game of go,” ICGA Journal, vol. 30, no. 4, pp. 198-208, 2007.
10. P. Baudiš and J. Gailly, “Pachi: State of the art open source Go program,” Advances in Computer Games, pp. 24-38, 2011.
11. D. Silver, et al., “Mastering the game of Go with deep neural networks and tree search,” Nature, vol. 529, no. 7587, pp. 484-489, 2016.
12. D. Silver, et al., “Mastering the game of Go without human knowledge,” Nature, vol. 550, no. 7676, pp. 354-359, 2017.
13. D. Silver, et al., “A general reinforcement learning algorithm that masters chess, shogi, and Go through self-play,” Science, vol. 362, no. 6419, pp. 1140-1144, 2018.
14. G. C. Stockman, “A minimax algorithm better than alpha-beta?,” Artificial Intelligence, vol. 12, no. 2, pp. 179-196, 1979.
15. M. Saks and A. Wigderson, “Probabilistic Boolean decision trees and the complexity of evaluating game trees,” in 27th Annual Symposium on Foundations of Computer Science (sfcs 1986), IEEE, 1986.
16. M. S. Campbell and T. A. Marsland, “A comparison of minimax tree search algorithms,” Artificial Intelligence, vol. 20, no. 4, pp. 347-367, 1983.
17. A. A. Elnaggar, et al., “A comparative study of game tree searching methods,” International Journal of Advanced Computer Science and Applications, vol. 5, no. 5, pp. 68-77, 2014.
18. J. Eisert, M. Wilkens, and M. Lewenstein, “Quantum games and quantum strategies,” Physical Review Letters, vol. 83, no. 15, p. 3077, 1999.
19. J. Du, et al., “Experimental realization of quantum games on a quantum computer,” Physical Review Letters, vol. 88, no. 13, p. 137902, 2002.
20. L. K. Grover, “A fast quantum mechanical algorithm for database search,” *Proceedings of the twenty-eighth annual ACM symposium on Theory of Computing*, 1996, pp. 212-219.
21. A. Ambainis, “Quantum search algorithms,” ACM SIGACT News, vol. 35, no. 2, pp. 22-35, 2004.
22. R. Cleve, D. Gavinsky, and D. L. Yonge-Mallo, “Quantum algorithms for evaluating min-max trees,” *in Workshop on Quantum Computation, Communication, and Cryptography,* Berlin, Heidelberg, 2008, pp. 11-15.
23. H. Barnum and M. Saks, “A lower bound on the quantum query complexity of read-once functions,” Journal of Computer and System Sciences, vol. 69, no. 2, pp. 244-258, 2004.
24. A. M. Childs, et al., “Exponential algorithmic speedup by a quantum walk,” in Proceedings of the thirty-fifth annual ACM symposium on Theory of computing, 2003.
25. A. M. Childs, “Universal computation by quantum walk,” Physical Review Letters, vol. 102, no. 18, p. 180501, 2009.
26. A. Ambainis, et al., “Any AND-OR formula of size N can be evaluated in time N^1/2+o(1) on a quantum computer,” SIAM Journal on Computing, vol. 39, no. 6, pp. 2513-2530, 2010.
27. D. A. Meyer and T. G. Wong, “Connectivity is a poor indicator of fast quantum search,” Physical Review Letters, vol. 114, no. 11, p. 110503, 2015.
28. C.-X. Weng, et al., “Beating the fault-tolerance bound and security loopholes for Byzantine agreement with a quantum solution,” Research, vol. 6, p. 0272, 2023.
29. B. W. Reichardt, “Faster quantum algorithm for evaluating game trees,” in Proceedings of the Twenty-Second Annual ACM-SIAM Symposium on Discrete Algorithms, Jan. 2011, pp. 546-559.
30. S. Chakraborty, et al., “Spatial search by quantum walk is optimal for almost all graphs,” Physical Review Letters, vol. 116, no. 10, p. 100501, 2016.
31. E. Farhi, J. Goldstone, and S. Gutmann, “A Quantum Algorithm for the Hamiltonian NAND Tree,” *Theory Comput,* vol. 4, pp. 169-190, 2007.
32. Y. Zhang, et al., “A New Type of Classical Logic Circuit with Exponential Speedup,” Advanced Intelligent Systems, vol. 5, no. 3, pp. 2200232, 2023.
33. N. Pan et al., “Electric-circuit realization of fast quantum search,” Research, 2021.
34. S. Liu, et al., “Topologically protected edge state in two-dimensional Su–Schrieffer–Heeger circuit,” Research, 2019.
35. H. Zhang, et al., “Electric-Circuit Simulation of Quantum Fast Hitting with Exponential Speedup,” Advanced Quantum Technologies, vol. 5, no. 4, p. 2100143, 2022.
36. S. Liu, et al., “Topologically protected edge state in two-dimensional Su–Schrieffer–Heeger circuit,” Research, 2019.
37. K. Luo, R. Yu, and H. Weng, “Topological nodal states in circuit lattice,” Research, 2018.
38. X. Tong, et al., “Quantum Combinational Logics and their Realizations with Circuits,” Advanced Quantum Technologies, vol. 7, no. 1, pp. 2300251, 2024.
39. P. W. K. Jensen, et al., “Molecular Realization of a Quantum NAND Tree,” *Quantum Science and Technology*, vol. 4, no. 1, pp. 015013, 2019.
40. Y. Wang, et al., “Integrated quantum-walk structure and NAND tree on a photonic chip,” *Physical Review Letters*, vol. 125, no. 16, pp. 160502, 2020.
41. Cao, W., et al., “Fully Integrated Parity–Time-Symmetric Electronics,” *Nature Nanotechnology*, vol. 17, no. 3, pp. 262-268, 2022.
42. Deng, W., et al., “High-order non-Hermitian topological electronics for ultra-sensitive integrated circuit sensors”, *Science Advances*, accepted, 2024
